# Multi-Frequency GPR Microwave Imaging of Sparse Targets through a Multi-Task Bayesian Compressive Sensing Approach

**DOI:** 10.3390/jimaging7110247

**Published:** 2021-11-21

**Authors:** Marco Salucci, Nicola Anselmi

**Affiliations:** Department of Civil, Environmental and Mechanical Engineering, DICAM, ELEDIA Research Center (ELEDIA@UniTN-University of Trento), Via Mesiano 77, 38123 Trento, Italy; nicola.anselmi.1@unitn.it

**Keywords:** inverse scattering (*IS*), microwave imaging (*MI*), ground penetrating radar (*GPR*), multi-frequency (*MF*), multi-task Bayesian compressive sensing (*MT-BCS*)

## Abstract

An innovative inverse scattering (*IS*) method is proposed for the quantitative imaging of pixel-sparse scatterers buried within a lossy half-space. On the one hand, such an approach leverages on the wide-band nature of ground penetrating radar (*GPR*) data by jointly processing the multi-frequency (*MF*) spectral components of the collected radargrams. On the other hand, it enforces sparsity priors on the problem unknowns to yield regularized solutions of the fully non-linear scattering equations. Towards this end, a multi-task Bayesian compressive sensing (*MT-BCS*) methodology is adopted and suitably customized to take full advantage of the available frequency diversity and of the a-priori information on the class of imaged targets. Representative results are reported to assess the proposed *MF-MT-BCS* strategy also in comparison with competitive state-of-the-art alternatives.

## 1. Introduction

During the last decades, many efforts have been devoted to the development of microwave imaging (*MI*) techniques for retrieving reliable and easy-to-interpret images of subsurface regions starting from the radargrams collected above the interface with a ground penetrating radar (*GPR*) [1,2,3,4,5,6,7,8,9]. The solution of the arising subsurface inverse scattering (*IS*) problem poses several challenges, mainly related to the intrinsic non-linearity (*NL*) and the ill-posedness (*IP*) [10]. On the one hand, the *NL* can be avoided by introducing Born-like approximations of the scattering equations [5], provided that weak scatterers are at hand and assuming that qualitative guesses (i.e., location and shape) are sufficient for the targeted application. Otherwise, multi-resolution strategies, integrated with both deterministic [7,9] and stochastic [8] optimization techniques, proved to be effective in mitigating the *NL* by reducing the ratio between unknowns and non-redundant informative data. On the other hand, the *IP* issue can be tackled by collecting the maximum amount of information from the scattering experiments. For instance, the wide-band nature of *GPR* measurements above the interface [1] provides an intrinsic frequency diversity in the collectable data. Such an information on the scenario under test can be profitably exploited with both frequency-hopping (*FH*) [6,7] and multi-frequency (*MF*) [8,9] *MI* techniques by processing each spectral component in a cascaded fashion or jointly, respectively.

Another effective recipe against the *IP* is the use of the *a-priori* information on the class of imaged targets. As a matter of fact, compressive sensing (*CS*)-based techniques [6,11,12,13,14] faithfully retrieved sparse objects (i.e., objects representable with few non-null expansion coefficients with respect to a suitably-chosen representation basis). Recently, *CS*-based methods have been proposed mainly to address and enhance the data acquisition speed in stepped frequency continuous wave (*SFCW*) *GPR*s [15,16,17,18]. Moreover, several deterministic *CS* strategies have been proposed for the tomographic imaging of buried targets that are intrinsically sparse with respect to the imaged domain including, for instance, landmines [19].

In such a framework, Bayesian *CS* (*BCS*) solvers have emerged as effective, computationally-fast, and also feasible tools since they do not require the compliance of the scattering operator with the restricted isometry property (*RIP*), whose check is often computationally unaffordable [11].

Following this line of reasoning, this paper presents a novel *MF* approach for reliably, robustly, and efficiently solving the *GPR-MI* of pixel-sparse subsurface objects. The proposed approach is based on a fully non-linear contrast source (*CSI*) formulation of the scattering equations, then solved by means of a customized multi-task *BCS* (*MT-BCS*) solver [13,14] based on a joint marginal likelihood maximization strategy that enforces the correlation between multi-static/multi-view wide-band *GPR* data. Therefore, to the best of the authors’ knowledge, the main novelty of this work relies on the development of a novel subsurface *IS* methodology combining the regularization capabilities of the *BCS* with those arising from the joint processing of multi-chromatic data. Accordingly, paramount challenges in *GPR-MI* are addressed related to (*i*) the implementation of an effective strategy counteracting the strong *IP* of the subsurface *IS* problem, (*ii*) the quantitative imaging of the subsurface domain to yield easy-to-interpret guesses of its *EM* composition, and (*iii*) the solution of the arising *MF-CSI* problem with high computational efficiency.

## 2. Mathematical Formulation

**Figure 1:**
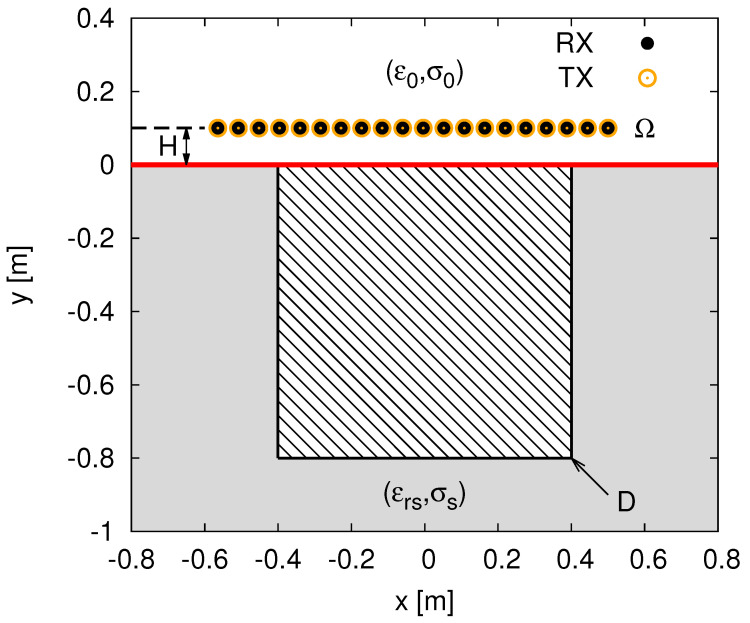
Geometry of the *2D-TM GPR-MI* problem.

Let us consider a two-dimensional transverse magnetic (*2D-TM*) half-space scenario where the investigation domain *D* is a subsurface region within a lossy soil with relative permittivity $\varepsilon_{rs}$ and conductivity $\sigma_{s}$ (Figure 1).

By considering a multi-static/multi-view measurement system, *D* is illuminated by *Vz*-oriented line sources placed in an observation domain Ω at distance *H* above the interface (Figure 1). The *v*-th (v=1,…,V) total electric field measured in time-domain by the *m*-th (m=1,…,M; $M=\left(V-1\right)$) receiver in Ω, ${\underline{r}}_{m}^{v}$ (${\underline{r}}_{m}^{v}=\left(x_{m}^{v},\;y_{m}^{v}=H\right)$), at the time-instant *t* (0≤t≤T), is given by [1]

(1) $$e^{v}\left({\underline{r}}_{m}^{v},t\right)=e_{i}^{v}\left({\underline{r}}_{m}^{v},t\right)+e_{s}^{v}\left({\underline{r}}_{m}^{v},t\right)$$

where $e_{i}^{v}$ and $e_{s}^{v}$ are the incident and scattered fields, respectively, while *T* is the duration of the *GPR* probing window. Being

(2) $$\Delta f=\left(f_{\max}-f_{\min}\right)$$

the 3 dB bandwidth of the transmitted waveform, the scattered field at the *p*-th (p=1,…,P) frequency,

(3) $$f_{p}=f_{\min}+\frac{\left(p-1\right)\Delta f}{\left(P-1\right)}$$

turns out to be

(4) $$E_{s,p}^{v}\left({\underline{r}}_{m}^{v}\right)=\Phi_{p}\left\{e^{v}\left({\underline{r}}_{m}^{v},t\right)-e_{i}^{v}\left({\underline{r}}_{m}^{v},t\right)\right\};\;\;\;\;\;m=1,\ldots ,M;\;v=1,\ldots ,V$$

where $\Phi_{p}\left\{a\left(\underline{r},t\right)\right\}=\int_{-\infty }^{\infty }a\left(\underline{r},t\right)\exp\left(-j2\pi f_{p}\right)dt$ is the Fourier transform. Moreover, it is related to the contrast function,

(5) $$\tau_{p}\left(\underline{r}\right)=\left[\varepsilon_{r}\left(\underline{r}\right)-\varepsilon_{rs}\right]+j\left[\frac{\sigma_{s}-\sigma \left(\underline{r}\right)}{2\pi f_{p}\varepsilon_{0}}\right]$$

modeling the unknown dielectric distribution in the investigation domain *D* at frequency $f_{p}$ (p=1,…,P), by the data equation [1]

(6) $$E_{s,p}^{v}\left({\underline{r}}_{m}^{v}\right)=\int_{D}G_{p}^{v}\left({\underline{r}}_{m}^{v},\underline{r}\right)J_{p}^{v}\left(\underline{r}\right)d\underline{r}$$

where $G_{p}^{v}\left({\underline{r}}_{m}^{v},\underline{r}\right)$ is the half-space Green’s function [1], while

(7) $$J_{p}^{v}\left(\underline{r}\right)=\tau_{p}\left(\underline{r}\right)E_{p}^{v}\left(\underline{r}\right)=\tau_{p}\left(\underline{r}\right)\Phi_{p}\left\{e^{v}\left(\underline{r},t\right)\right\}$$

is the *v*-th (v=1,…,V) equivalent current induced within the investigation domain.

### Inverse Problem Solution Approach

To numerically solve the inverse problem at hand, the Equation (6) is first recast into the following matrix expression

(8) $${\underline{\xi }}_{p}^{v}={\underline{\underline{\Psi }}}_{p}^{v}{\underline{\upsilon }}_{p}^{v}$$

by partitioning *D* into *N* square sub-domains centered at $\left\{{\underline{r}}_{n};\;n=1,\ldots ,N\right\}$ so that

(9) $${\underline{\xi }}_{p}^{v}={\left[ℜ\left({\underline{E}}_{s,p}^{v}\right),\;ℑ\left({\underline{E}}_{s,p}^{v}\right)\right]}^{T}$$

being ${\underline{E}}_{s,p}^{v}=\left\{E_{s,p}^{v}\left({\underline{r}}_{m}^{v}\right);\;m=1,\ldots ,M\right\}$,

(10) $${\underline{\upsilon }}_{p}^{v}={\left[ℜ\left({\underline{J}}_{p}^{v}\right),\;ℑ\left({\underline{J}}_{p}^{v}\right)\right]}^{T}$$

being ${\underline{J}}_{p}^{v}=\left\{J_{p}^{v}\left({\underline{r}}_{n}\right);\;n=1,\ldots ,N\right\}$, and

(11) $${\underline{\underline{\Psi }}}_{p}^{v}=\left[\begin{array}{cc} ℜ\left({\underline{\underline{G}}}_{p}^{v}\right) & -ℑ\left({\underline{\underline{G}}}_{p}^{v}\right)\\ ℑ\left({\underline{\underline{G}}}_{p}^{v}\right) & -ℜ\left({\underline{\underline{G}}}_{p}^{v}\right) \end{array}\right],$$

${\underline{\underline{G}}}_{p}^{v}$ being the $\left(v,p\right)$-th (v=1,…,V; p=1,…,P) $\left(M\times N\right)$ half-space Green’s matrix [1], while $.^{T}$ stands for the transpose operator and $ℜ\left(\;.\;\right)/ℑ\left(\;.\;\right)$ denotes the real/imaginary part.

Successively, the solution of (8) is found with a customized multi-frequency multi-task *BCS* (*MF-MT-BCS*) technique [14] by jointly enforcing the spatial sparsity of the unknown components of the equivalent currents

(12) $$\left\{{\underline{\upsilon }}_{p}^{v};\;v=1,\ldots ,V;\;p=1,\ldots ,P\right\}$$

(${\underline{\tilde{\upsilon }}}_{p}^{v}=\left\{{\tilde{\upsilon }}_{p,n}^{v};\;n=1,\ldots ,2N\right\}$; ${\tilde{\upsilon }}_{p,n}^{v}=ℜ\left({\tilde{J}}_{p}^{v}\left({\underline{r}}_{n}\right)\right)$ and ${\tilde{\upsilon }}_{p,\left(n+N\right)}^{v}=ℑ\left({\tilde{J}}_{p}^{v}\left({\underline{r}}_{n}\right)\right)$), and their correlation among the different illuminations and spectral components, the number of “tasks” solved in parallel being equal to $L=\left(V\times P\right)$. More specifically, the *v*-th (v=1,…,V) equivalent current at the *p*-th (p=1,…,P) frequency is computed as

(13) $${\underline{\tilde{\upsilon }}}_{p}^{v}={\left[\mathrm{diag}\left(\underline{\tilde{\alpha }}\right)+{\left({\underline{\underline{\Psi }}}_{p}^{v}\right)}^{†}{\underline{\underline{\Psi }}}_{p}^{v}\right]}^{-1}{\left({\underline{\underline{\Psi }}}_{p}^{v}\right)}^{†}{\underline{\xi }}_{p}^{v}$$

by applying a fast relevant vector machine (*RVM*) method [14] to solve the following optimization problem

(14) $$\underline{\tilde{\alpha }}=\arg\left\{\max_{\underline{\alpha }}\left[-0.5\sum_{p=1}^{P}\sum_{v=1}^{V}\left(2M+2\delta_{1}\right)\log\left[{\left({\underline{\xi }}_{p}^{v}\right)}^{†}{\left({\underline{\underline{U}}}_{p}^{v}\right)}^{-1}{\underline{\xi }}_{p}^{v}+2\delta_{2}\right]+\log\left|{\underline{\underline{U}}}_{p}^{v}\right|\right]\right\}$$

for retrieving the set of 2N hyper-parameters $\underline{\alpha }=\left\{\alpha_{n};\;n=1,\ldots ,2N\right\}$ shared among the *V* views and *P* frequencies. In (14),

(15) $${\underline{\underline{U}}}_{p}^{v}=\underline{\underline{I}}+{\underline{\underline{\Psi }}}_{p}^{v}{\left[\mathrm{diag}\left(\underline{\alpha }\right)\right]}^{-1}{\left({\underline{\underline{\Psi }}}_{p}^{v}\right)}^{†},$$

$\underline{\underline{I}}$ being the identity matrix, is an auxiliary matrix analogous to (14) in [14], while $\delta_{1}$ and $\delta_{2}$ are *BCS* control parameters. Moreover, . ${}^{†}$ and $\left|\;.\;\right|$ indicate the conjugate transpose and the determinant, respectively.

Finally, the contrast distribution (n=1,…,N) at the central frequency,

(16) $$f_{c}=\frac{\left(f_{\min}+f_{\max}\right)}{2}$$

is derived as

(17) $$\tilde{\tau }\left({\underline{r}}_{n}\right)=\frac{1}{P}\sum_{p=1}^{P}\left(ℜ\left\{{\tilde{\tau }}_{p}\left({\underline{r}}_{n}\right)\right\}+j\frac{f_{p}}{f_{c}}ℑ\left\{{\tilde{\tau }}_{p}\left({\underline{r}}_{n}\right)\right\}\right)$$

where

(18) $${\tilde{\tau }}_{p}\left({\underline{r}}_{n}\right)=\frac{1}{V}\sum_{v=1}^{V}\frac{{\tilde{J}}_{p}^{v}\left({\underline{r}}_{n}\right)}{{\tilde{E}}_{p}^{v}\left({\underline{r}}_{n}\right)}$$

${\tilde{J}}_{p}^{v}\left({\underline{r}}_{n}\right)={\tilde{\upsilon }}_{p,n}^{v}+j{\tilde{\upsilon }}_{p,\left(n+N\right)}^{v}$ and ${\tilde{E}}_{p}^{v}\left({\underline{r}}_{n}\right)$ being the $\left(v,p\right)$-th (v=1,…,V; p=1,…,P) retrieved current and the corresponding total electric field in the *n*-th (n=1,…,N) cell of the investigation domain (${\underline{r}}_{n}\in D$), respectively.

## 3. Numerical Assessment

To assess the proposed *MF-MT-BCS* approach, representative numerical results are shown and discussed in this Section. A square investigation domain *D* of side 0.8 m buried in a medium with $\varepsilon_{rs}=4.0$ and $\sigma_{s}=10^{-3}$ (S/m) [9] has been considered as a reference benchmark scenario. Moreover, a set of V=20 sources and $M=\left(V-1\right)=19$ probes (for each illumination), located in an observation domain Ω placed at H=0.1 m above the interface (Figure 1), has been chosen for the sensing setup to collect the time-domain *GPR* radargrams. These latter have been simulated with the *GPRMax2D* SW [20], while the scattered spectrum has been sampled at P=9 uniformly-spaced frequencies within the 3 dB band $\left(f_{\max},\;f_{\min}\right)=\left(200,\;600\right)$ MHz (Δf=400 MHz, $f_{c}=400$ MHz of the Gaussian monocycle excitation centered at $f_{0}=300$ MHz [6]. As for the setting of the *MF-MT-BCS* control parameters (14), the optimal trade-off values $\left(\delta_{1},\;\delta_{2}\right)=\left(6\times 10^{-1},\;9\times 10^{-5}\right)$ have been derived from a preliminary calibration performed by blurring the time-domain total field data samples with different levels of white Gaussian noise. Finally, to provide a quantitative measure of the reconstruction accuracy, the following integral error has been considered [9]

(19) $$\Xi_{reg}=\frac{1}{N_{reg}}\sum_{n=1}^{N_{reg}}\frac{\left|\tau \left({\underline{r}}_{n}\right)-\tilde{\tau }\left({\underline{r}}_{n}\right)\right|}{\left|\tau \left({\underline{r}}_{n}\right)+1\right|}$$

where $\tau \left({\underline{r}}_{n}\right)$ and $\tilde{\tau }\left({\underline{r}}_{n}\right)$ denote the actual and retrieved contrast at frequency $f_{c}$, respectively, while “*reg*” indicates that the computation considers the whole imaged domain (“*reg*” ⇒ “*tot*”), the target support (“*reg*” ⇒ “*int*”), or the external background (“*reg*” ⇒ “*ext*”).

**Figure 2:**
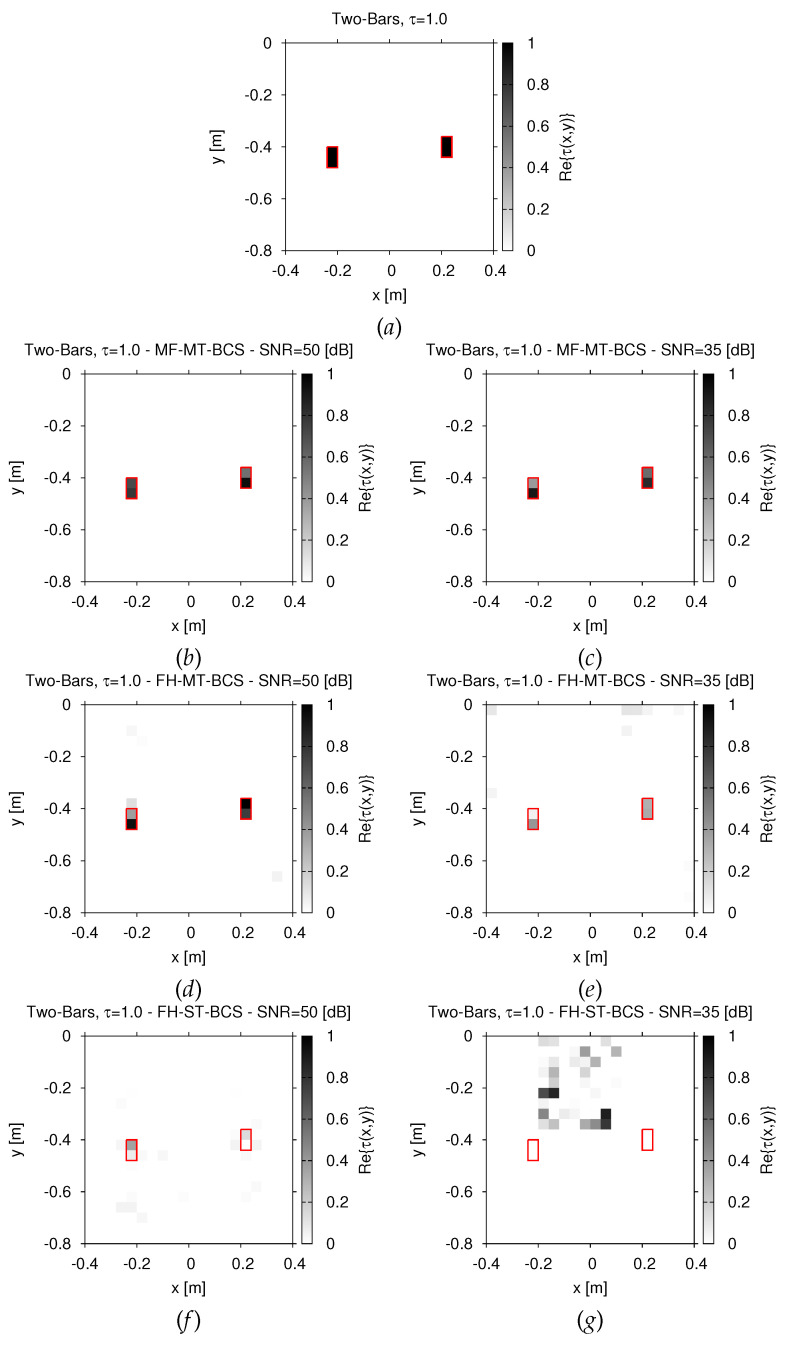
*Numerical assessment* (“*Two-Bars*” *Scatterer*, τ=1.0, N=400)—Actual (**a**) and retrieved (**b**–**g**) dielectric profile by (**b**,**c**) the *MF-MT-BCS*, (**d**,**e**) the *FH-MT-BCS*, and (**f**,**g**) the *FH-ST-BCS* when processing noisy data at (**b**,**d**,**f**) SNR=50 dB and (**c**,**e**,**g**) SNR=35 dB.

The first test case is concerned with the “*Two-Bars*” scattering profile of Figure 2a ($\varepsilon_{r}=5$, $\sigma =10^{-3}$ (S/m) ⇒τ=1.0). The *MF-MT-BCS* data inversion gives a very accurate image of *D* independently on the data signal-to-noise ratio (*SNR*) and it faithfully recovers the support, as well as the contrast value of the two buried scatterers (Figure 2b,c vs. Figure 2a).

**Figure 3:**
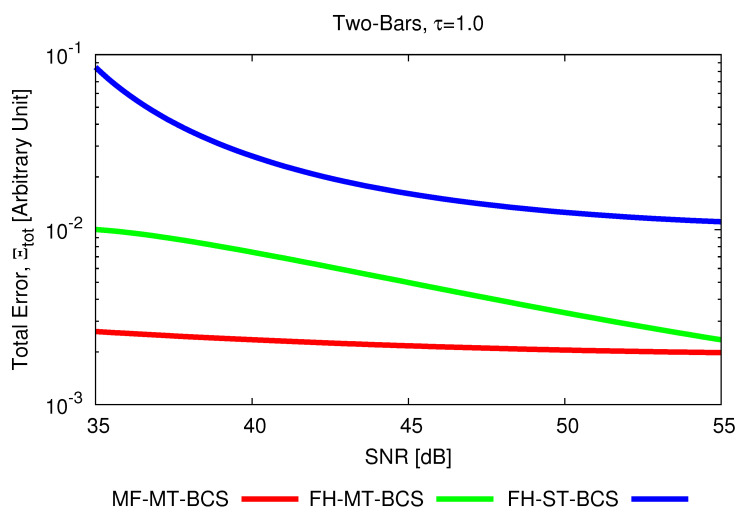
*Numerical assessment* (*“Two-Bars” Scatterer*, τ=1.0, N=400, $SNR\in \left[35,\;55\right]$ dB)—Behavior of the total integral error as a function of the *SNR* on time-domain total field for the *MF-MT-BCS*, *FH-MT-BCS*, and *FH-ST-BCS* methods.

To better point out the advantage of jointly processing all spectral components of the scattered field, as done by the proposed *MF* inversion scheme, the results of two *FH*-based state-of-art *BCS* solution strategies are reported in Figure 2 for comparison purposes. It is worthwhile to remind oneself that these methods process each *p*-th (p=1,…,P) frequency in a cascaded fashion, from the lowest to the highest one, by either enforcing the correlation between multiple views (L=V tasks—*FH-MT-BCS* method [6]) or considering each view as a single task (L=1—*FH-ST-BCS* method [6]). As it can be observed, the *MF-MT-BCS* outperforms both *FH* strategies, the worst inversion being performed by the *FH-ST-BCS* (Figure 2f,g). Such outcomes are quantitatively confirmed by the values of the total error, $\Xi_{tot}$, reported in Figure 3 versus the *SNR*. The *MF-MT-BCS* does not only provide the lowest errors, but it is also significantly more robust against the data noise since, for instance, $\frac{{\left.\Xi_{tot}\right|}_{MF-MT-BCS}^{SNR=35\;[\mathrm{dB}]}}{{\left.\Xi_{tot}\right|}_{FH-MT-BCS}^{SNR=35\;[\mathrm{dB}]}}=2.5\times 10^{-1}$ (Figure 2e vs. Figure 2c) and $\frac{{\left.\Xi_{tot}\right|}_{MF-MT-BCS}^{SNR=35\;[\mathrm{dB}]}}{{\left.\Xi_{tot}\right|}_{FH-ST-BCS}^{SNR=35\;[\mathrm{dB}]}}=3.0\times 10^{-2}$ (Figure 2g vs. Figure 2c) in the most critical working conditions (i.e., SNR=35 dB on time-domain total field).

**Figure 4:**
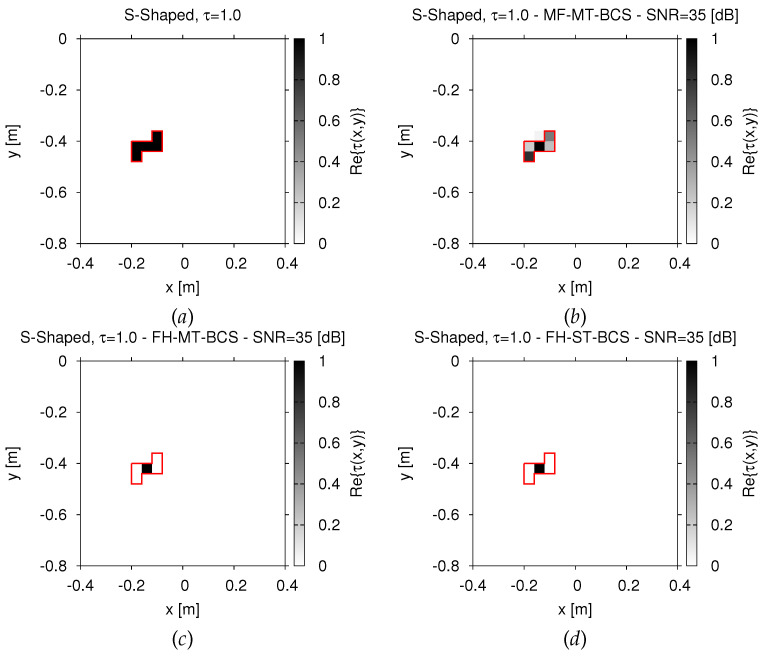
*Numerical assessment* (“*S-Shaped*” *Scatterer*, τ=1.0, N=400, SNR=35 dB)—Actual (**a**) and retrieved (**b**,**d**) dielectric profile by (**b**) the *MF-MT-BCS*, (**c**) the *FH-MT-BCS*, and (**d**) the *FH-ST-BCS*.

Similar conclusions can be drawn also when dealing with a more complex-shaped scatterer. As a matter of fact, the “*S-shaped*” object (τ=1.0, Figure 4a) has been imaged by the *MF-MT-BCS* (Figure 4b vs. Figure 4a) remarkably better than the *FH-MT-BCS* (Figure 4c) and the *FH-ST-BCS* (Figure 4d), both *FH* methods failing in retrieving the actual support of the scatterer.

**Figure 5:**
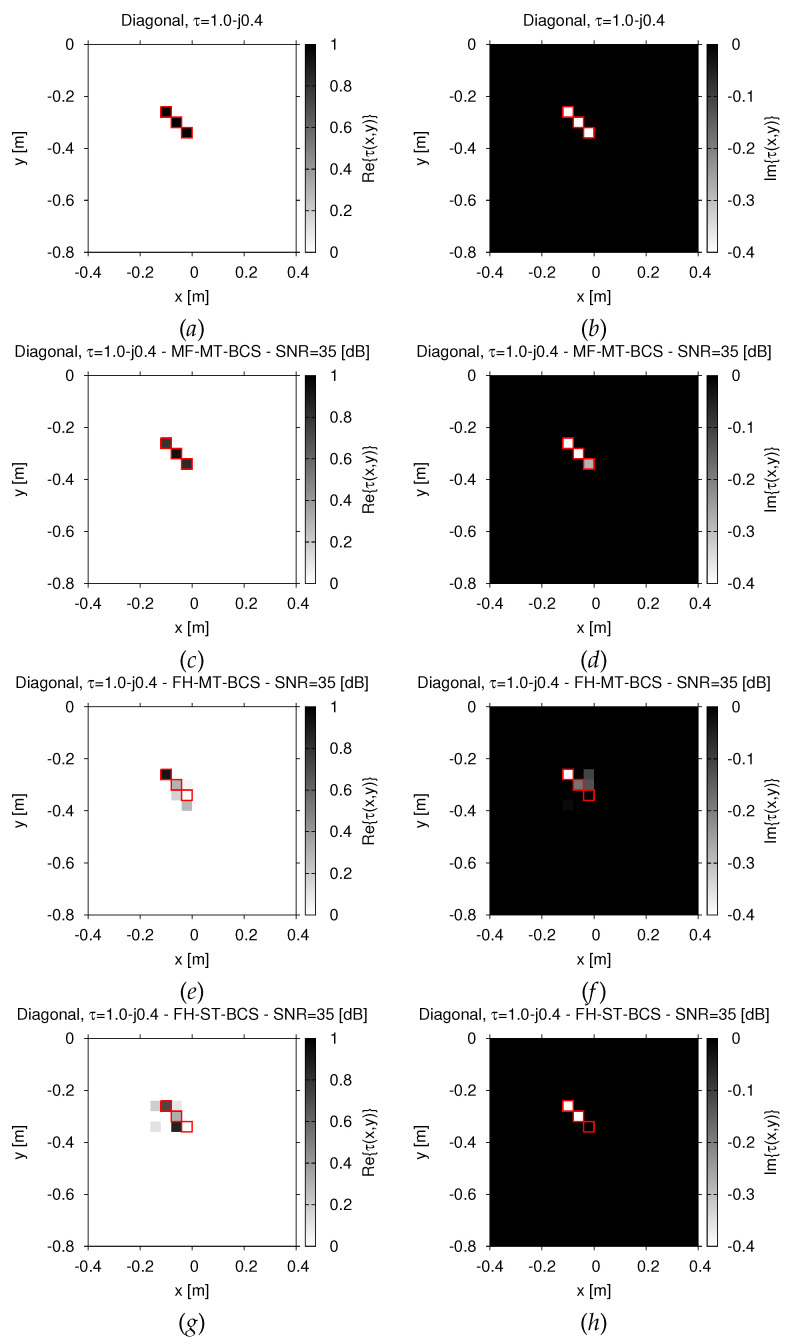
*Numerical assessment* (“*Diagonal*” *Scatterer*, τ=1.0−j0.4, N=400, SNR=35 dB)—Actual (**a**,**b**) and retrieved (**c**–**h**) real part (**a**,**c**,**e**,**g**) and imaginary part (**b**,**d**,**f**,**h**) of the contrast outputted by (**c**,**d**) the *MF-MT-BCS*, (**e**,**f**) the *FH-MT-BCS*, and (**g**,**h**) the *FH-ST-BCS*.

The *MF-MT-BCS* is more effective to recover objects with a higher conductivity than the hosting medium, as well. Indeed, despite the increased complexity due to the presence of a non-null imaginary part of the contrast and the non-negligible amount of noise, it is the only method able to provide an accurate guess of both the real part (Figure 5c vs. Figure 5a) and the imaginary one (Figure 5d vs. Figure 5b) of the “*Diagonal*” scatterer ($\varepsilon_{r}=5$, $\sigma =10^{-2}$ (S/m) ⇒ τ=1.0−j0.4—Figure 5a,b). In addition to the pictorial representations in Figure 5, the performance of each inversion method have been quantified in terms of the total, the internal (i.e., within the target support), and the external (i.e., in the background) errors [9], the corresponding values being reported in Table 1. Finally, it is worth pointing out the higher efficiency exhibited by the *MF-MT-BCS* thanks to the “one-shot” inversion of all *P* frequency components of the *GPR* spectrum. As a representative example, let us consider that the reduction in the inversion time on a standard laptop with Intel(R) Core(TM) i5-8250U CPU @ 1.60GHz and 16 (GB) of RAM memory amounts to $\frac{{\left.\Delta t\right|}_{FH-MT-BCS}}{{\left.\Delta t\right|}_{MF-MT-BCS}}=22.1$ and $\frac{{\left.\Delta t\right|}_{FH-ST-BCS}}{{\left.\Delta t\right|}_{MF-MT-BCS}}=85.8$, respectively (Table 1).

Having assessed the superior performance of the *MT-MT-BCS* over the two *FH*-based methods, the last set of numerical benchmarks is aimed at verifying the robustness of the proposed *IS* strategy when taking into account the effects of soil inhomogeneity and non-planar air–soil interfaces.

**Figure 6:**
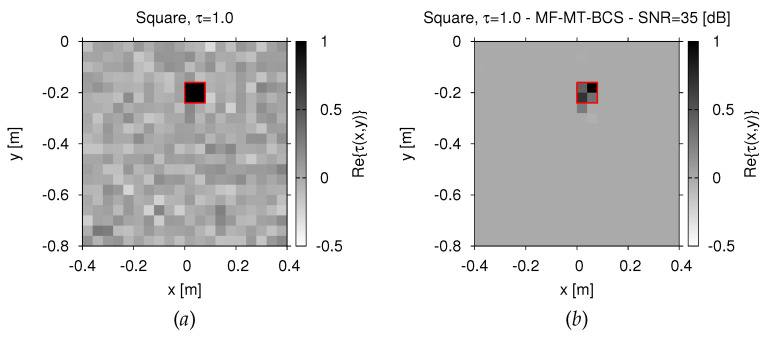
*Numerical assessment* (“*Square-Shaped*” *Scatterer*, τ=1.0, N=400, SNR=35 dB)—Actual (**a**) and retrieved (**b**) dielectric profile by the *MF-MT-BCS* when considering a randomly-varying inhomogeneous soil permittivity.

Towards this end, a square-shaped target with τ=1.0 has been imaged when considering a uniform random perturbation of the soil permittivity within the range $\varepsilon_{rs}\in \left[3.5,\;4.5\right]$, as shown in Figure 6a. Despite the higher complexity of the problem at hand and the non-negligible amount of noise on measurements, the retrieved contrast distribution in Figure 6a indicates that the *MF-MT-BCS* is capable of retrieving the unknown target with a remarkable accuracy, as also quantitatively verified by the computed internal error ($\Xi_{int}=2.17\times 10^{-1}$).

**Figure 7:**
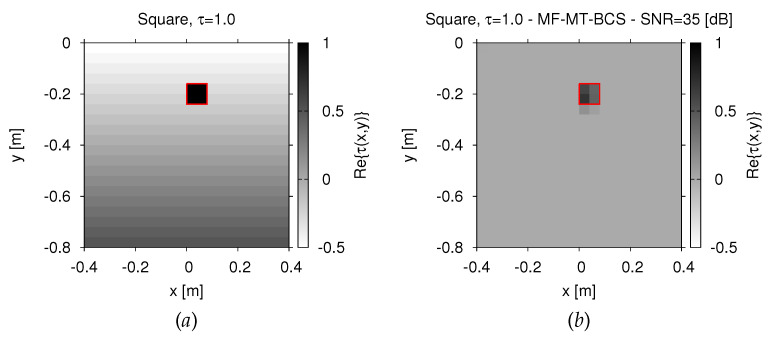
*Numerical assessment* (“*Square-Shaped*” *Scatterer*, τ=1.0, N=400, SNR=35 dB)—Actual (**a**) and retrieved (**b**) dielectric profile by the *MF-MT-BCS* when considering a smoothly-varying inhomogeneous soil.

Similar outcomes are obtainable also when dealing with a smoothly-varying background medium [7], as shown in Figure 7a. As a matter of fact, the plot of the retrieved dielectric profile in Figure 7b indicates that the performance of the *MF-MT-BCS* is not jeopardized in such operative conditions, with $\Xi_{int}=2.33\times 10^{-1}$.

**Figure 8:**
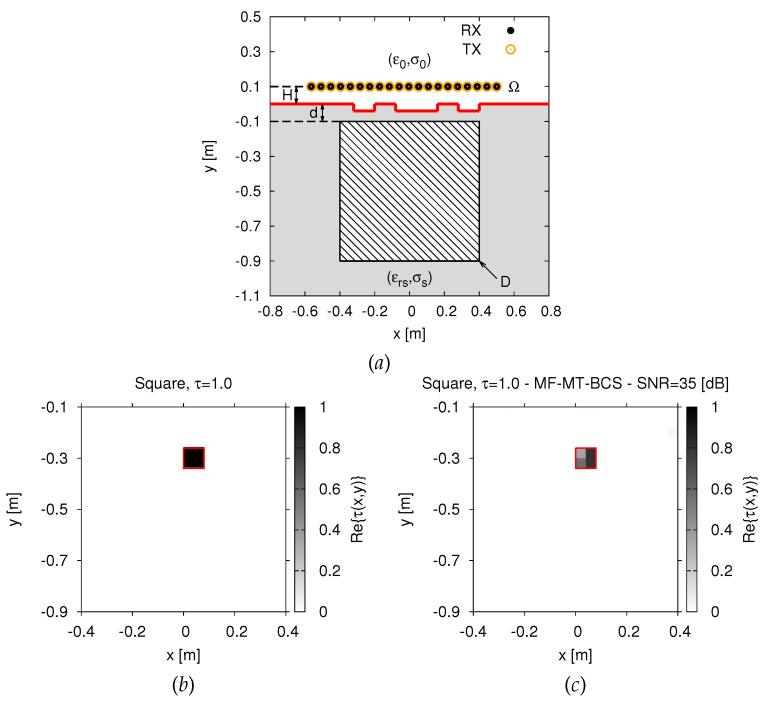
*Numerical assessment* (“*Square-Shaped*” *Scatterer*, τ=1.0, N=400, SNR=35 dB)—Imaging scenario (**a**) and actual (**b**) and retrieved (**c**) dielectric profile by the *MF-MT-BCS* when considering a non-planar air–soil interface.

The *MF-MT-BCS* exhibits a high robustness also when the air–soil interface is not planar as in previous test cases. To prove it, let us consider in the following the subsurface scattering scenario depicted in Figure 8a, where random perturbations of the air–soil interface have been considered when imaging an investigation domain *D* buried at a depth of d=0.1 m. Regardless of the presence of the non-planar interface (not modeled by the considered half-space Green’s operator used in the inversion [1]), a highly accurate guess of the actual target has been yielded by the *MF-MT-BCS* ($\Xi_{int}=1.88\times 10^{-1}$—Figure 8c vs. Figure 8b).

## 4. Conclusions

A novel sparsity-promoting strategy has been proposed to effectively solve the *2D GPR-MI* problem. Thanks to the adopted *MF* strategy, the *MF-MT-BCS* method allows a computationally-efficient exploitation of the frequency-diversity of the *GPR* data by correlating all the multi-chromatic components extracted from the measured radargrams. As a result, it outperforms available *FH*-based solution strategies formulated within the *BCS* framework by exhibiting remarkably higher accuracy, robustness, and computational efficiency. It is worth pointing out that the main assumption for the successful application of the proposed method is that the unknown targets are *intrinsically* sparse (i.e., they are small-sized with respect to the investigation domain *D* or, in other words, their support occupies a low percentage of total number of pixels, *N*). However, depending on the targeted application, such a limitation can be easily overcome by exploiting other (i.e., non-pixel) representations allowing to represent the problem unknowns with few non-null expansion coefficients and thus enabling a proficient application of the *CS* paradigm [21].

## Figures and Tables

**Table 1:** *Numerical assessment* (“*Diagonal*” *Scatterer*, τ=1.0−j0.4, N=400, SNR=35 dB)—Total, internal, and external reconstruction errors [9] and inversion time for the *MF-MT-BCS*, the *FH-MT-BCS*, and the *FH-ST-BCS* methods.

	*MF-MT-BCS*	*FH-MT-BCS*	*FH-ST-BCS*
$\Xi_{tot}$	$5.94\times 10^{-4}$	$6.11\times 10^{-3}$	$1.09\times 10^{-2}$
$\Xi_{int}$	$7.92\times 10^{-2}$	$3.37\times 10^{-1}$	$3.57\times 10^{-1}$
$\Xi_{ext}$	0.0	$3.60\times 10^{-3}$	$8.24\times 10^{-3}$
Δt (s)	3.1	68.5	266

## Data Availability

Not applicable.
